# Declining pre-monsoon dust loading over South Asia: Signature of a changing regional climate

**DOI:** 10.1038/s41598-017-16338-w

**Published:** 2017-11-22

**Authors:** Satyendra K. Pandey, V. Vinoj, K. Landu, S. Suresh Babu

**Affiliations:** 1 School of Earth, Ocean and Climate Sciences, Indian Institute of Technology Bhubaneswar, Odisha, 752 050 India; 2 Space Physics Laboratory, Vikram Sarabhai Space Centre, Trivandrum, Kerala 695022 India

## Abstract

Desert dust over the Indian region during pre-monsoon season is known to strengthen monsoon circulation, by modulating rainfall through the elevated heat pump (EHP) mechanism. In this context, an insight into long term trends of dust loading over this region is of significant importance in understanding monsoon variability. In this study, using long term (2000 to 2015) aerosol measurements from multiple satellites, ground stations and model based reanalysis, we show that dust loading in the atmosphere has decreased by 10 to 20% during the pre-monsoon season with respect to start of this century. Our analysis reveals that this decrease is a result of increasing pre-monsoon rainfall that in turn increases (decreases) wet scavenging (dust emissions) and slowing circulation pattern over the Northwestern part of the sub-continent.

## Introduction

Mineral dust is among dominant natural aerosol species in the atmosphere, besides sea salt^1^ and is generated due to wind erosion over arid and semiarid regions of the globe. It affects the Earth system through variety of processes. One such process is scattering and absorption of solar and terrestrial radiation, known as the direct radiative effect^2–4^. Another is by modulating cloud characteristics^5–8^, which alters radiative properties of cloud, known as the semi-direct and indirect radiative effects^9,10^. Besides important role in the atmospheric processes, deposition of dust due to long range transport on the glaciers are also found to have profound impact on the planetary albedo^11,12^ and hence the radiation budget^13^. Dust also modulates atmospheric dynamics^14–16^ through warming within the atmosphere thereby altering circulation patterns. Other than its effect on various components of the climate system^17–20^, mineral dust also has many other impacts, such as on marine productivity through nutrient deposition^21^, deterioration of air quality by contributing to particulate matter PM2.5^22^, on human health by causing acute respiratory diseases^23–26^. The studies in the past have shown that the presence of large absorbing dust in the atmosphere can enhance the lower atmospheric warming^27,28^. For example, it has been shown that Indian summer monsoon rainfall is related to dust loading (both locally and remotely) at different time scales^16,27,29–31^. The elevated heat pump hypothesis^27^ states that pre-monsoon dust from local sources combined with black carbon could alter the rainfall during the early part of the monsoon season over the central Indian region. It is also shown that large loading of aerosols could potentially alter the north-south sea-surface temperature gradient thereby altering the strength of monsoon circulation. In addition, the eastward flow of dust aerosols could also reduce the surface reaching solar irradiance^32–34^ and potentially modulate local circulation patterns. The mineral dust is also found to have an impact on Himalayan glaciers^35^ during pre-monsoon by darkening of surface snow cover. Dust also affects atmospheric chemistry by mediating heterogeneous chemical reaction through surface adsorption^36^ and plays an important role in global biogeochemical cycle^37,38^.

The aerosol burden over India is about three times higher than the global mean values due to abundance of mineral dust, especially during pre-monsoon and monsoon seasons^34,39^. The production of mineral dust is sensitive to synoptic conditions, winds, precipitation and surface characteristics^40–42^. However, the recent changes to land-sea temperature contrast^43^, due to anthropogenic climate forcing, may have also led to alteration in aforementioned parameters, which in turn may affect dust abundance in the atmosphere^44^. Past studies using long term surface measurements as part of ARFINET^45^ and AERONET^46^ site at Kanpur have shown that on an annual basis, aerosol optical depth is increasing at a rate of ~3.0% per year^47,48^ over the past few decades. This is not surprising knowing the fact that the rapid development in the economy of the country and associated activities are expected to increase emissions and hence aerosol loading in the atmosphere. However, seasonal trends in aerosol loading are inconclusive^45–49^ during pre-monsoon and monsoon when dust loading is highest. The increasing trend is mostly attributed to increasing economic activity and consequent emissions. Also, focus in the recent times is mostly on changes to anthropogenic activities and subsequent aerosol loading^45–48^ and their effects. However, not much attention is given to changes, if any, specifically on natural aerosols like dust over the land.

For instance, it may be noted that the increases in anthropogenic emissions are most prominent during the post-monsoon and the winter season, whereas rainfall mostly occurs during the monsoon season. Though there are model based hypothesis relating anthropogenic aerosols to monsoon rainfall^50–52^, there is observational evidence only relating absorbing dust aerosols and monsoon rainfall at different time scales^16,31,53^ with some uncertainties with respect to the physical mechanisms. Therefore, it is critical to understand changes in dust emissions over this region especially during the monsoon and the pre-monsoon periods. In this study, we investigate whether there are any changes to dust aerosol loading in the atmosphere in the recent decades during the pre-monsoon season. If yes, what factors led to these changes? It may be noted that such analysis were not possible due to paucity of reliable, high quality, long term measurements with sufficient spatial and temporal resolutions until recently where ARFINET, IMD-SKYNET and AERONET sites have expanded to cover vast areas of the country. Also, dedicated satellite based aerosol measurements (MODIS and MISR) and surface based inversion products were only available since year 2000. We have therefore, synergistically used these publically available surface and satellite measurements along with chemical reanalysis products (from MERRA2) to understand how aerosols, and specifically dust loading has changed over the Indian region during the pre-monsoon season and explore its potential causes.

## Results and Discussion

The atmospheric aerosol loading over the Indian region peaks during the pre-monsoon (March to May) and the monsoon (June to August) seasons depending on location of interest and the spatial distribution of rainfall^49,54–56^. However, during the monsoon season, rainfall virtually removes aerosols from the atmosphere through wet scavenging leading to low loading conditions immediately after rainfall. Also, the errors associated with the analysis of aerosol trends during monsoon season using satellite measurements are exacerbated by both cloud contamination^57^ and the inter-relationship between rainfall and aerosol loading^49,58^. Therefore, in this study we restrict our analysis to the pre-monsoon period, thereby maximizing (minimizing) the clear sky conditions (rainfall and/or cloud contamination if any). The organization of this manuscript is as follows. First, we determine the trend in AOD over the Indo-Gangetic Plain (IGP) using the longest available high quality dataset from multiple AERONET sites. We then carry out similar analysis on aerosol properties from multiple satellites. We compare the results from surface observations with features captured by the satellites. Once, we confirm that the changes are consistent; we then determined whether these changes are due to dust that in turn changes composite AOD loading, using a widely used simple decision tree classification method. We thereafter reconfirm these findings using chemical reanalysis datasets available for dust loading. Finally, we study the potential causes that led to changes in dust loading over the study region during the analysis period (2000 to 2015) using multiple satellite precipitation and reanalysis products. We then briefly discuss the potential implication of these findings.

### Changes in column aerosol optical depth

#### Ground based Measurements

Recent studies have shown that aerosol loading over the Indian region is increasing on an annual mean basis^45,47^. However, it is found that these trends have marked differences seasonally. These changes have been attributed or speculated to various causes including both natural and anthropogenic activities/sources^45,46,59^. Past studies were mostly based on station datasets with limited spatial coverage. There were no independent species specific datasets available to determine whether these long term changes are caused by dust or anthropogenic loading as the primary datasets were mostly column AOD and their spectral dependence. Therefore, our first objective was to investigate changes occurring during the pre-monsoon season when dust loading is the highest using ground stations (AERONET sites) across the Northern Indian subcontinent.

**Figure 1:**
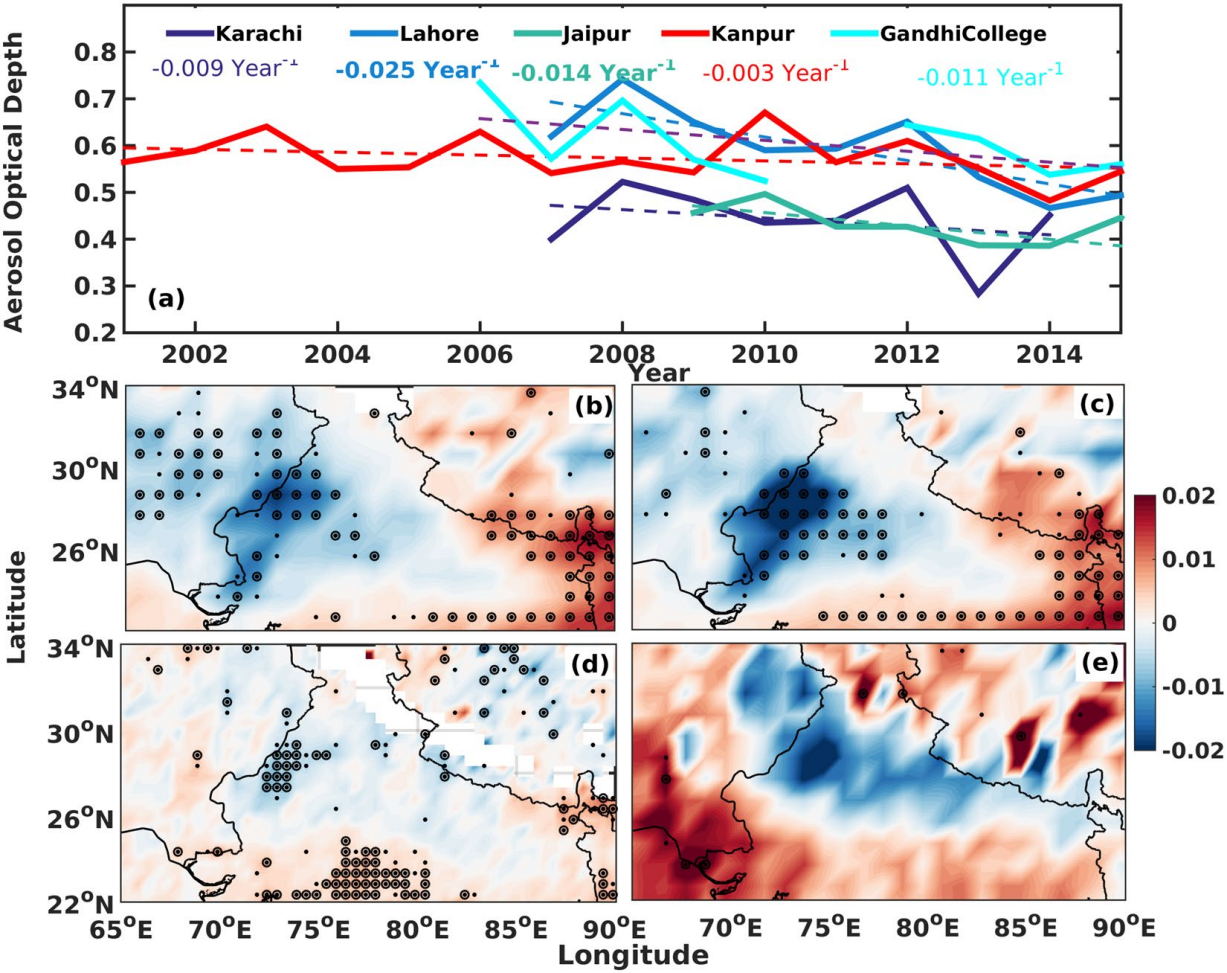
(**a**) The trend (year^−1^) of seasonal mean aerosol optical depth over the AERONET sites in the IGP for pre-monsoon season. The bold text indicates significance at 90% confidence level. (**b**) to (**e**) shows the spatial pattern of trends (year^−1^) in aerosol optical depth observed using different sensor/satellite platforms (**b**) MODIS Terra & (**c**) MODIS Aqua combined Deep Blue Dark Target, (**d**) MISR and (**e**) OMI-UV Aerosol Index. The black dots (circle) represent statistical significance at 90% (95%) confidence level. The map was generated using MATLAB 2015b, www.mathworks.com.

Our analysis reveals that almost all the ground based sites (see Fig. 1), with sufficiently long time series data of at least a decade, show a decreasing trend in the column aerosol loading during pre-monsoon period. Also, the decrease is most pronounced over the sites located to the west of the Indo-Gangetic Plains with a clear East-West gradient. Lahore and Jaipur, to the west of the IGP/North India show highest decrease (0.025 and 0.014 per year respectively, which is ~3% per year with respect to year 2000) in the column loading. On the other hand, Kanpur, a site at center of the IGP shows comparatively lesser decrease (0.003 per year, ~0.5%). Moreover, the decrease in AOD is even prominent over the site Gandhi College, the eastern part of the IGP. This shows that the decrease is widespread during the pre-monsoon season over the whole greater IGP belt. However, the largest decreases were observed over Jaipur and Lahore. It may however be noted, that annually, each of these surface sites showed an increasing trend (mostly attributable to anthropogenic activities) in aerosol loading^45,46,60^. Therefore, this decrease observed during the pre-monsoon period may mostly be a consequence of something related to the natural activities, most probably due to dust aerosols considering the locations of maximum change and its close proximity to the Thar Desert. However, there are no independent data on dust concentration/emission to verify this. Also, the gradient mentioned based on surface measurements need to be independently verified to see any large scale change. We therefore, used observation from multiple platforms (both ground and space based) along with model/reanalysis based simulations to assess whether there have been changes to dust aerosol loading and if yes, what are the potential factors that contributed to this change.

#### Satellite based measurements

The trend analysis were carried out using satellite based measurements from different platforms (MODIS Terra, MODIS Aqua, MISR and OMI) and different retrieval methodologies (Dark Target (DT) approach^61^, Deep Blue (DB)^62^ and combined Dark Target and Deep Blue (DT & DB)) at each grid point (at both 0.5 and 1.0 degree resolutions) over the Indian region. It may be noted that AOD retrievals using a total of eight different retrieval methodologies are used in this study. We find that there is a significant and wide spread (large spatial extent covering the North Western part of Indian subcontinent) decreasing trend in aerosol loading during the pre-monsoon period (Fig. 1b to e). The black dots (circle) represent statistical significance at 90% (95%) confidence level. The East-West gradient in ground based measurements is found to be quite evident here. Nevertheless, it may be noted that the Eastern regions still shows a clear increasing trend in the aerosol loading. However, the largest decreasing trend is found over the Thar Desert and the arid regions to the west.

Consistent results obtained using both ground (five sites) and satellite (eight retrieval methods) based measurements provide us confidence that these changes are not due to retrieval errors (differing methodologies) or platform specific drifts (in each of the sensors) that may lead to erroneous trends^63^. Though these observations indicate that the aerosol loading and thereby concentration in the atmosphere may be decreasing, there are no independent and validated satellite based methodology to verify the type of aerosol that is changing. However, the aerosol index from OMI is shown to be sensitive to absorbing dust aerosols^64^ and this shows similar decreasing trend as well. This provides indication that it may be the dust and/or black carbon aerosols that is decreasing.

**Figure 2:**
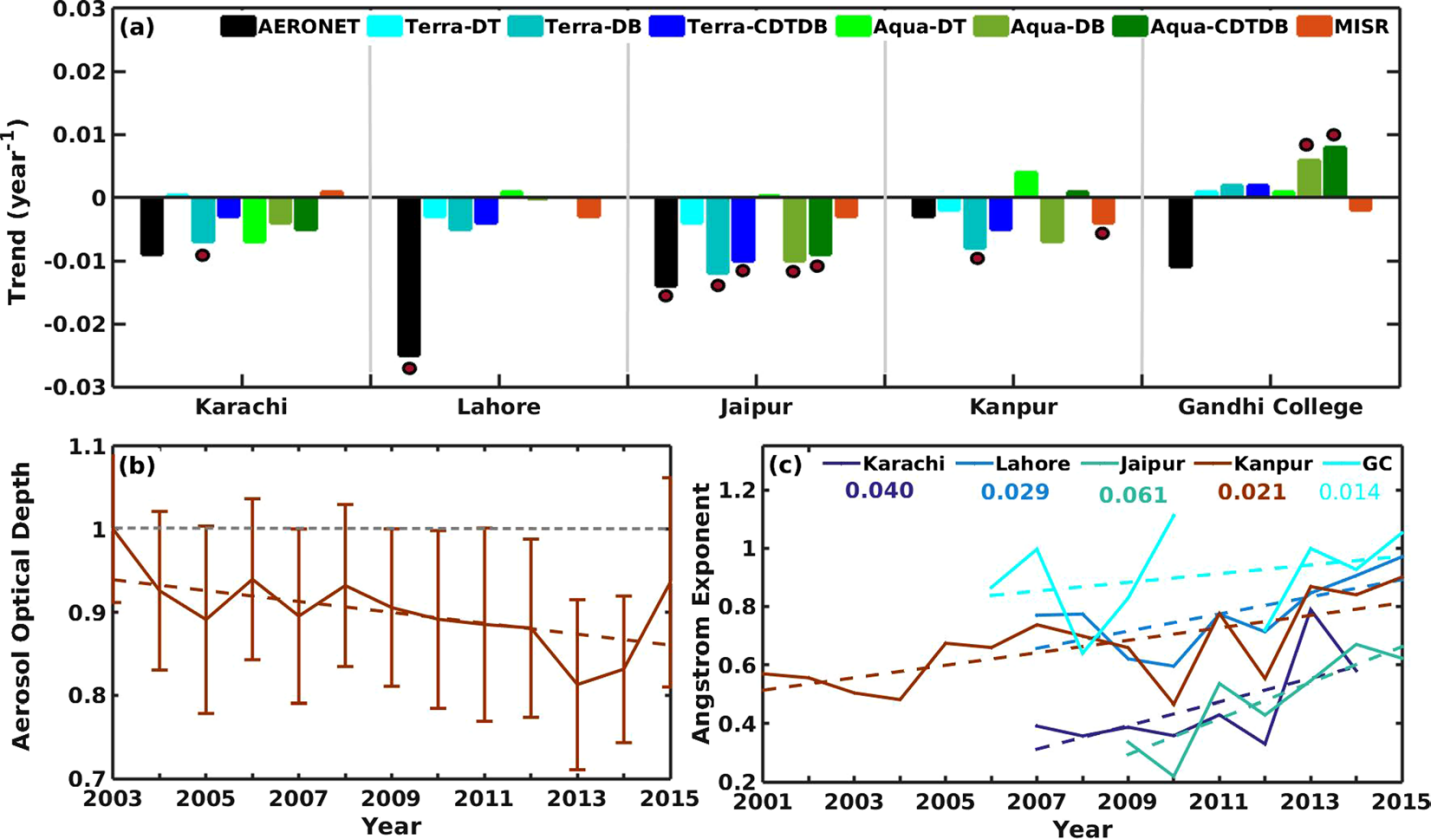
Inter annual trend (year^−1^) in (**a**) AOD from satellite and ground based data (2001 to 2015) for Stations over IGP, the dots represent statistical significance at the 90% confidence level (**b**) mean AOD (normalized with initial value) obtained from all the satellite data used in the present study over five sites, error bars represents the 95th percentile of inter-site and inter-sensor differences and (**c**) angstrom exponent (α_440–870_), bold font indicates statistical significance at the 90% confidence level. The map was generated using MATLAB 2015b, www.mathworks.com.

Figure 2a summarizes, changes observed using the ground based AERONET sites over the IGP along with similar analysis based on different satellite measurements (based on different retrievals and platforms). All show similar results, albeit slight changes in their magnitudes. A clear east-west gradient is also observed in the trends with larger decreases in AOD to the West. Though ground based measurements showed a decreasing trend over Gandhi College (the eastern most site), none of the MODIS retrievals showed a decreasing trend for Gandhi College, which is the East most site among the sites used in our study. However, both ground based AERONET and MISR (0.5 × 0.5 degree) showed a consistent decreasing trend. It is not clear, what could have caused this, but we speculate it to be a bias created by the use of coarser resolution (1 × 1 degree) MODIS that is representative of a larger region around the ground site. In addition, Fig. 2b shows the mean trend from all satellite sensors and ground stations (normalized with respect to 2003), which clearly exhibits the decreasing trend. It may be noted that even the 95^th^ percentile of the data for all sites and satellites lie below the base year mean.

The possibility for the east-west gradient in trends could be the decrease in dust contribution away from dust sources (to the West), which renders anthropogenic aerosols to dominate the fractional contribution to the total loading and hence trends (smaller decreases) in the Eastern part of IGP. It may also be noted that different retrieval methodologies have different assumptions, complexities and differing levels of sophistication. It is therefore difficult to narrow down the exact reason for slight differences in trends between sensors and retrieval methodologies. Overall majority of the sensors and methodologies agree to the declining aerosol loading over the different sites with a clear east-west gradient.

It may also be mentioned that there are no indications showing that other types of aerosols such as from anthropogenic sources (such as Black Carbon) are decreasing. It may be noted that if BC were decreasing (increasing), it will strengthen (weaken) the trends. However in this study, we focus our attention on dust as its contribution to total AOD is several fold larger than other aerosols over this region. On a cursory note, the increasing economic activities and resultant anthropogenic emissions render the possibility of a decrease in BC a remote possibility. This is also evident from the trend in wavelength dependence of spectral AOD (i.e. in Angstrom Exponent (α_440–870_, AE) observed in the AERONET data showing a clear increase over the years during the pre-monsoon season (see Fig. 2c). Similar results were observed for spatial variability in AE (see Fig. S1). However, increasing AE could be due to either, increasing smaller particles (perhaps of anthropogenic origin) or decreasing larger particles (natural dust).

Having determined that the decrease in AOD is tracked by both surface and satellite based sensors and the spatial pattern indicating that the decrease is mostly to the west of IGP and over the Thar Desert. We therefore use a simple methodology to confirm whether the changes are due to dust. It may be noted that the observed spatial (west to east) gradient is only an indication and is not an evidence that dust is decreasing during the study period.

#### Observed change in dust from ground based measurements

In order to determine what type of aerosol have changed and to confirm our hypothesis on dust change, we used a decision tree based methodology developed by Lee *et al*., 2010^65^. The methodology is employed using long term surface measurements of both extinction and absorption, extracted from AERONET data, in order to determine the dominant aerosol types and their changes. The types classified are carbonaceous (absorbing fine-mode), soil dust (absorbing coarse-mode), sulphate (non-absorbing fine-mode), and sea-salt (non-absorbing coarse-mode) particles. This methodology has widely been used to segregate aerosol types based on ground based measurements^66,67^ over IGP. We find that the method is also able to capture the seasonality in aerosol types over the Indian region with dust dominating during pre-monsoon and monsoon period transitioning to black carbon during winter (see Fig. S2). This observed seasonality is in agreement with previous studies e.g.^68–73^. In this study, we specifically focus only on the classification related to dust.

**Figure 3:**
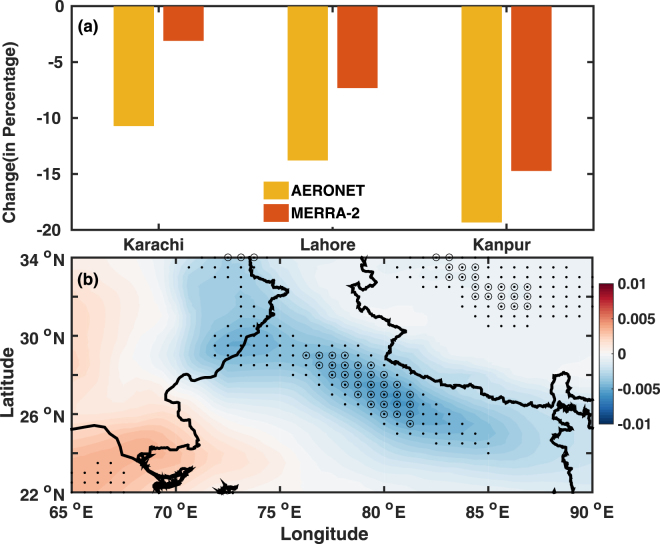
The changes in dust observed over the Northern part of the sub-continent (**a**) the percentage change in dust loading during pre-monsoon season (between the period 2011–2014 and 2006–2010) using an independent analysis to identify dust loading using AERONET retrieved aerosol size information and absorption. (**b**) MERRA2 reanalysis for the period 2002 to 2015 (year^−1^). The black dots (circle) represent statistical significance at 90% (95%) level. The map was generated using MATLAB 2015b, www.mathworks.com.

Based on the longest available data source, we have chosen Karachi, Kanpur and Lahore AERONET sites for the change analysis. Incidentally these sites are also located at two extreme sides of the IGP. Our analysis (Fig. 3a) reveals that there has been significant reduction in dust loading over the period of our study. The decreases are between 10 and 20% over all the sites. The percentage decreases are more pronounced over Kanpur, in the Eastern part, than over Lahore, which is closer to the dust source regions. This may be due to the difference in lower frequency of occurrence and/or dust loading over Kanpur (in comparison to cities close to desert source) thereby showing a higher % value of change over the East than over the West, where frequency of occurrence of dust events are higher. This analysis provides us the confidence/evidence that the decrease observed in column loading based on ground and satellite based measurements is due to changes in dust aerosols. We also used MERRA2 retrieved dust AOD to derive the trends during the study period (Fig. 3b). It is found that there exists a clear decreasing trend in dust AOD with a stronger gradient towards the west (Fig. 3b). This finding further provides us an additional indication that the observed change in AOD’s were due to dust and the maximum trends are over the dust source regions to the North-western part of India.

We carried out several analyses to confirm that aerosol type classification used in this study is robust. Our investigation (see Supplementary material) shows that errors (as per AERONET retrieval) associated with either absorption or extinction (is <5%) does not affect our finding with respect to aerosol type identification (Fig. 3a). MERRA2 dust AOD also shows similar results albeit slight differences in magnitude. Having confirmed that the change in column loading is due to change in dust using both ground based measurements and MERRA2 reanalysis dust AOD, we proceed to investigate the potential cause for the observed change.

### Potential cause for decrease in dust loading

The aerosol loading over any location is a function of the strength of local source, sink and advection (long range transport). Therefore, changes to both rainfall and circulation patterns could have played a significant role in the observed changes to dust over the Indian region. Increased rainfall over the source region will alter dust concentration/loading in two ways, one by increasing wet removal and other by altering the emission strength due to changes in soil moisture, which in turn alters the erodibility. Therefore, even minor changes to rainfall over dust source regions could have large effect on column dust loading. We therefore used Tropical Rainfall Measuring Mission (TRMM), Global Precipitation Climatology Project (GPCP), University of Delaware gridded Precipitation (UDel) and India Meteorology Department (IMD) gridded rainfall datasets to analyse the precipitation changes over the study domain.

**Figure 4:**
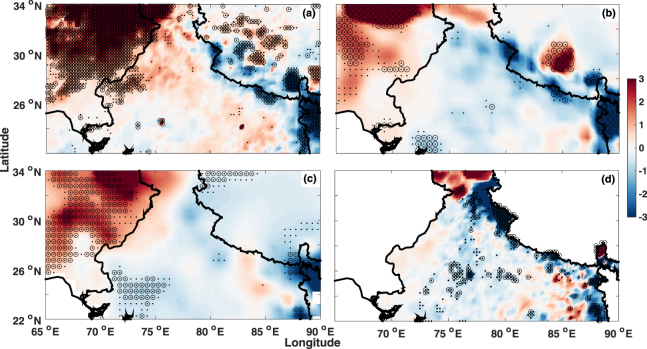
Spatial pattern of rainfall trends (year^-1^) from different datasets (**a**) TRMM (**b**) GPCP (**c**) UDel and (**d**) IMD. The black dots (large dots) indicate 90% (95%) confidence level. The map was generated using MATLAB 2015b, www.mathworks.com.

Our analysis (Fig. 4) reveals that the pre monsoon rainfall has increased during this period with maximum increase observed over the Pakistan region and also over the Thar Desert. The rainfall increase over Thar Desert is not significant for datasets using station based datasets. This could be a result of issues related to number of stations over the desert region going in to the production of these gridded data products such as IMD, UDel and GPCP. However, TRMM which directly obtains the spatial pattern (at each grid point) shows the increases in rainfall over the Thar Desert region. Overall, it is found that the rainfall is increasing over the arid and semi-arid regions to the NW part of the sub-continent.

**Figure 5:**
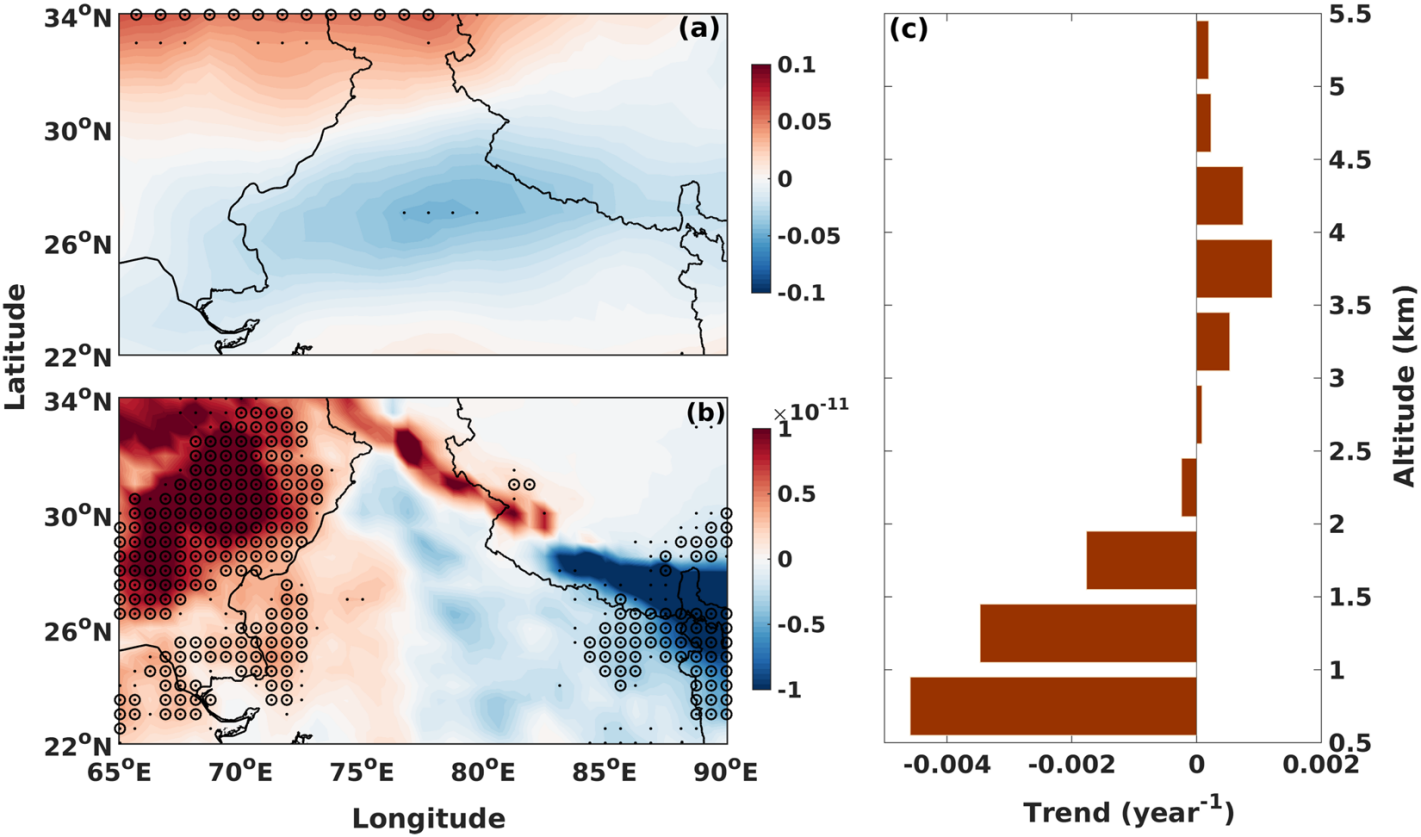
The spatial pattern of trends (year^−1^) in (**a**) 10 m wind speed (ms^−1^) (ECMWF-ERA-Interim) (**b**) Dust wet deposition (kg m^−2^s^−1^) (MERRA-2). The black dots (circle) represent statistical significance at 90% (95%) confidence level. (**c**) Trend in Extinction Coefficient (km^−1^) observed from the CALIPSO data over Jaipur (close to the dust source regions) during the period 2006 to 2015. The plots were generated using MATLAB 2015b, www.mathworks.com

In addition, any change in circulation that may alter the winds over the source region will also alter the strength of dust emission. We therefore calculated the trend in 10 m wind speed obtained from ECMWF–ERA reanalysis. There is a clear indication of a slowdown of winds (see Fig. 5a) especially in the vicinity of Thar Desert. This in turn may reduce local emissions of dust loading. Similar observations were also made over African region^74^. This, in addition, will also slow down any dust transport away from the desert regions. The increased rainfall also increased the wet scavenging (see Fig. 5b). Based on the above observations, we hypothesise that major factors that contributed to pre-monsoon decreasing trends in aerosols over Indian region are, (i) Increased rainfall in the western part of Indian subcontinent that removed aerosol loading through wet scavenging including the dust aerosols. (ii) Reduced emission of dust due to increased precipitation which decreased the erodibility and hence dust emission. (iii) The slowing winds further reduced the efficiency of long range transport thereby decreasing loading over regions away from the dust sources. Even the dust that were generated will not be transported longer distances if the winds are slowing.

Therefore, our analysis reveals that there is a clear decrease in AOD observed during pre-monsoon period over the Northern part of the Indian region and specifically over the North western part of Indian subcontinent due to declining dust emissions. This is due to the increased rainfall, which inhibited dust emission and at the same time increased wet scavenging. It may be mentioned that the role of rainfall on aerosol loading will be more evident if high resolution vertical distribution of aerosols are available. We therefore used the layer mean extinction coefficient at 532 nm from CALIPSO profiles nearest to Jaipur station located close to the Desert region to assess the change. As expected, our analysis (Fig. 5c) reveals that the reduction in aerosol loading is most concentrated below 2.5 km altitude. This is a clear evidence for the role of wet scavenging/removal and/or reduced emissions and therefore lower loading conditions in these altitudes. In addition, the slowing down of winds over this region also further plays a role in reducing the generation and thereafter long-range transport of dust aerosols, which were eventually picked up by sites far from the dust sources. However, this appears to be of minor importance compared to the role played by rainfall. It may be mentioned that factors contributing to change in observed rainfall and winds over this region are beyond the scope of this work and are not explored further. We have thus restricted out investigation to only the changes to dust and their immediate meteorological causes for declining trends during pre-monsoon season. Summarizing, our analysis using long term ground, multiple satellite and model based reanalysis products reveal that dust aerosol loading over Northern India has decreased by as much as 10 to 20% since year 2000, depending on location in the past 15 years due to increasing pre-monsoon rainfall and slowing atmospheric circulation pattern. This decrease in dust loading may also provide an additional feedback leading to increased irradiance over the region thereby strengthening the Pakistan low that influences the monsoon flow. These competing effects along with anthropogenic aerosols and changing climate may modify monsoon rainfall in different ways and need to be addressed so as to understand the effect of aerosols, and specifically dust, on monsoon rainfall. It may be mentioned that the above analysis and hence the conclusions are based primarily on observations arising out of multiple ground and satellite based measurements. Therefore, it primarily shows that the changing regional climate is modifying dust dynamics over the south Asian region. Further research is necessary based on regional climate modelling initiatives to understand the feedback induced by changes to dust cycle on regional climate over South Asia.

## Summary and Conclusion

Our analysis using multiple ground and satellite based measurements along with reanalysis products reveal that,

1. The atmospheric aerosol loading over Northern Indian subcontinent (especially IGP) is decreasing during pre-monsoon season.
2. This decrease is due to change in dust emission over the arid and desert regions over North western part of Indian subcontinent including Thar Desert.
3. The potential cause for this change is the changing regional climate induced increasing rainfall over North-western subcontinent which increased wet scavenging and decreased erodibility and hence dust emissions. In addition, the weakening pre-monsoon circulation pattern further reduced the emissions and long-range transport.

## Data and Methods

### Datasets

In the present study ground based aerosol parameters were obtained from AErosol RObotic NETwork (AERONET) sites located over Indo-Gangetic Plain viz., Kanpur, Gandhi College, Jaipur, Lahore and Karachi. Level 2.0 qualities assured, cloud screened direct and inversion products for all five stations were used. AERONET uses CIMEL sun photometer, which provides the spectral data of direct sun and sky radiances within the spectral range of 340–1020 nm and 440–1020 nm; however sky-radiances were obtained only at four channels. We have used spectral AOD at mid-visible (500 nm), Fine Mode Fraction (FMF) and derived Single Scattering Albedo (SSA). The accuracy in retrieved AOD is of the order of ±0.01(λ > 440 nm) to ±0.02(for λ < 440 nm); while sky radiance uncertainties is assumed to be within ±5% for all wavelengths^75^. FMF obtained using spectral de-convolution algorithm^74^ is well validated and quantitative measure of fine mode dominance in the total loading. It is better measurement of aerosol size than Angstrom exponent which is more qualitative.

Along with ground data set, we have used AOD obtained from satellite borne sensors such as Moderate Resolution Imaging Spectroradiometer (MODIS), Multi-Angle Imaging Spectro Radiometer (MISR) and Ozone Monitoring Instruments (OMI). The Moderate Resolution Imaging Spectroradiometer sensor (MODIS) is on board NASA’s Terra (since 2000), equator crossing time 10:30 A.M. local time, and Aqua (since 2002), equator crossing time of 1:30 P.M. local time, polar orbiting satellites. It provides long term aerosol observational data, suitable for trend analysis. In the present study the level 3 (Collection-6, MOD08_D3_v6 and MYD_08_D3_v6) aerosol optical depth at 550 nm with a spatial resolution of 1 degree is used. MODIS aerosol products have been validated extensively for quality. The retrieval algorithm has undergone continuous improvement by modification to cloud masking procedures employed, aerosol models and surface reflectance database used^76,77^. Multi-Angle Imaging Spectro Radiometer (MISR) flying onboard NASA’s Terra satellite is a multi-angular and multispectral sensor. It takes observations at nine viewing angles, with four spectral bands centered at 446 nm, 558 nm, 672 nm and 866 nm. In the present study, level- 3 (MIL3DAEv4) gridded AOD (555 nm) having spatial resolution of 0.5 degree is used. MISR-AOD values are well correlated and falls within 20% limit of AERONET-AOD^78^. Ozone Monitoring Instruments is onboard EOS-Aura (since 2004) Satellite. Aerosol Index derived from OMI measured radiance uses near ultraviolet algorithm^79^ and an indicator of absorbing aerosol such as dust and soot particles. We have used OMTOd3v003 level-3 global product having spatial resolution of 1 × 1 degree which uses only good quality level-2 products.

Apart from aerosol parameters we have used rainfall from Tropical Rainfall Measuring Mission (TRMM) and Global Precipitation Climatology Project (GPCP) to understand the impact of rainfall changes on the dust loading. The monthly TRMM precipitation data (TRMM_3B43v7) for the study period (2001–2016) with spatial resolution of 0.25 × 0.25 degree were obtained from GIOVANNI (https://giovanni.gsfc.nasa.gov/) online visualization system^80^. More details of TRMM products can be found in Huffman, 2015^81^. GPCP monthly precipitation dataset combines satellite and ground based observations available since 1979 at spatial resolution of 2.5 × 2.5 global grids. In this study we have used version2.3^82^. We have also used the gridded rainfall datasets from India Meteorological Department (IMD)^83^ and University of Delaware (UDel)^84^ version 4.01. In addition to above dust extinction AOD, dust wet and dry deposition data was obtained from MERRA-2. The NASA’s Modern-Era Retrospective analysis for Research and Applications version 2 (MERRA-2)^85^ provides global reanalysis product from 1980 to near real time. Aerosol products from MERRA-2 is extensively validated with independent observations^86,87^. ERA-Interim^88^ 10 m wind speed data is obtained from ECMWF were also used.

### Aerosol Type determination

A simple decision tree based algorithm developed by Lee 2010^65^, were used. Based on size and absorption characteristics aerosol type can be classified into four type fine mode absorbing, coarse mode absorbing, fine mode non-absorbing and coarse mode non-absorbing^65^. Dust can be identified as coarse mode absorbing aerosol. This method uses FMF and SSA as size and absorption measurement respectively. It in turns gives the dominance of particular type of aerosol for particular set of observations. We have chosen two sub-periods (viz., 2006–10 and 2011–14) based on data availability to identify the changes in dust loading. The dust extinction AOD obtained from MERRA2 was used to verify the obtained results.

### The calculation of aerosol trends

Seasonal average of daily mean AOD obtained from AERONET sites located over the IGP was used to calculate inter-annual trend of the column loading. A simple linear trend model was fitted and tested against parametric student t-test. Satellite observations were further examined to evaluate the spatial extent of these changes and relate it to any particular aerosol types. In addition to that same linear fit were also used for the trend analysis of rainfall and MERRA2 dust products. It may be mentioned that inversion noise in aerosol retrievals may pose a challenge in carrying out trend analysis using satellite retrievals especially when the changes studied are within the bounds of inversion noise of satellite retrievals. We carried out the trend analysis in each of station, satellite and reanalysis products by removing top and bottom five percentile of the data so as to remove any retrievals affected by thin clouds or stray light in to the sensor. The results were consistent with those reported in the manuscript. Our confidence in the trends also stems from the similarity in results from multiple line of evidence based on independent sources.

## Electronic supplementary material

Supplementary Material
