# Supplementary material for: Declining pre-monsoon dust loading over South Asia: Signature of a changing regional climate
Source: Sci Rep. 2017 Nov 22;7:16062. doi: 10.1038/s41598-017-16338-w (PMC5700173; doi:10.1038/s41598-017-16338-w)
Supplement: Supplementary file 1 — Supplementary Material [file 41598_2017_16338_MOESM1_ESM.pdf]

# Declining pre-monsoon dust loading over South Asia: Signature of a changing regional climate

Satyendra K. Pandey[1], V. Vinoj[1*], K. Landu[1] and S. Suresh Babu[2]

[1]School of Earth, Ocean and Climate Sciences Indian Institute of Technology Bhubaneswar Odisha, INDIA - 752 050.

[2]Space Physics Laboratory, Vikram Sarabhai Space Centre, Trivandrum, Kerala, INDIA-695022.

*Correspondence Email: vinoj@iitbbs.ac.in

## Trend in Angstrom Exponent

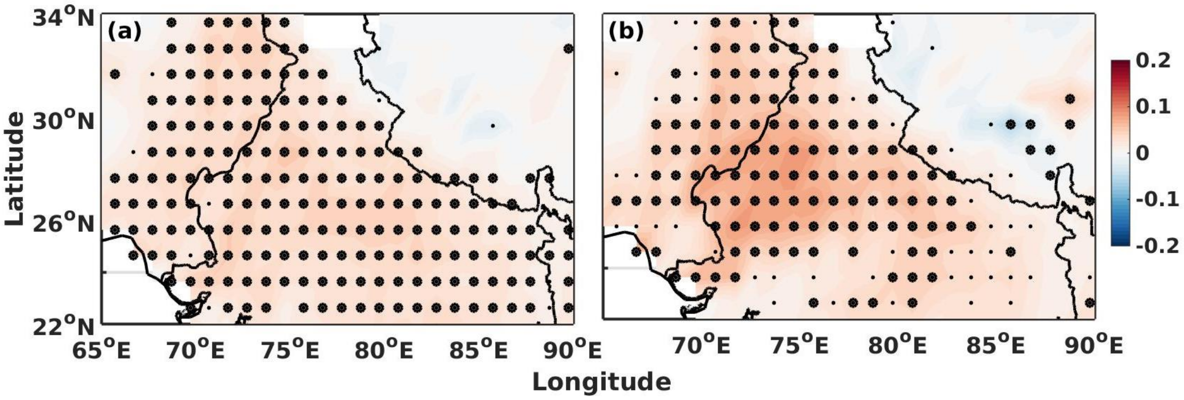


Fig S1: Spatial pattern of trend (year$^{-1}$) in Angstrom Exponent from MODIS (a) Terra and (b) Aqua. The large (small) dots represent statistical significance at the 95 % (90 %) confidence level. The map was generated using MATLAB 2015b, www.mathworks.com

## Aerosol Type Determination

The decision tree based algorithms provide one confirmed output per set of observations, thereby providing the best suitable aerosol type assigned to a set of observations, though other type of aerosols may also be present. Therefore, the compositions discussed here may be considered as frequency distribution of aerosol types. We have carried out the analysis using daily data and also all individual retrievals from AERONET during the study period. The results obtained were similar, no matter what data frequency is used. Also, sensitivity analysis was carried out on

different parameters (like single scattering albedo, SSA) and their sensitivity to the final outcome of aerosol types. This provides us the confidence that the methodology and analysis carried out are robust and consistent.

**Potential Errors**

In addition, we also investigated the possibility of misclassification of aerosol types associated with error in absorption and found it to be varying between dust and the uncertain aerosol type (not shown). This shows that dust aerosol type identification can only be misclassified if a consistent pattern of inverse relationship is found between dust and the uncertain aerosol type. Any decrease in dust must be seen as an increase in uncertain aerosol type and vice-versa if it were a misclassification. We do not find this pattern. Also, our analysis reveals that this misclassification amplifies only if error associated with SSA is more than 10 to 15%. (not shown). The standard error associated with AERONET retrieval of SSA is less than 5% [3,4], therefore potential for misclassification of dust is quite small and the reported analysis is robust. The above analysis provided us confidence that the results obtained are robust and are not due to analysis methodology.

**Seasonality in aerosol Type**

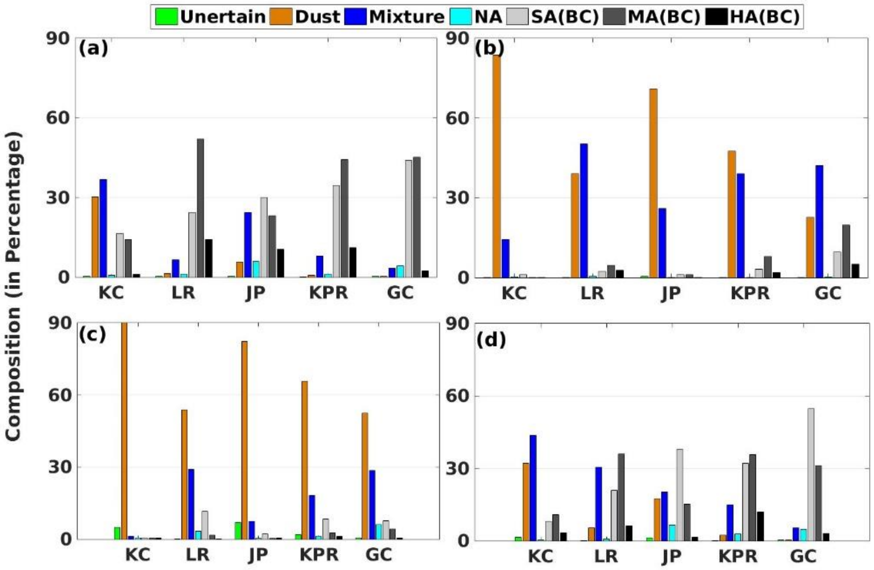


Fig S2: Seasonality in the observed aerosol type Five stations over IGP. (a)Winter (b) Pre-monsoon (c) Monsoon and (d) Post Monsoon . where, KC: Karachi , LR :Lahore, JP: Jaipur ,KPR-Kanpur and GC: Gandhi College . NA : Non absorbing, SA: slightly absorbing, MA: moderately absorbing HA: Highly absorbing BC: Black Carbon. The map was generated using MATLAB 2015b, www.mathworks.com

Fig S2 shows the observed seasonality in the aerosol type over Northern India. Dust is dominating aerosol type during pre-monsoon (Fig S1b) and monsoon (Fig S1c), while Black Carbon is in the post-monsoon and winter. The east-west gradient in aerosol type is also evident. It can be observed east-ward decrease in the dust and increase in black carbon is observed.

The above result, when placed along with multiple line of evidence from AERONET sites over IGP and multiple datasets from different satellites, shows conclusively that dust is decreasing over western part of IGP. In addition to the above analysis, we also used MERRA2 reanalysis datasets for dust AOD. We find a consistent decrease (see Fig. 5a in main text) in this dataset. This again confirms that dust indeed is decreasing over the IGP as a consequence of decreasing emissions over the Thar Desert.

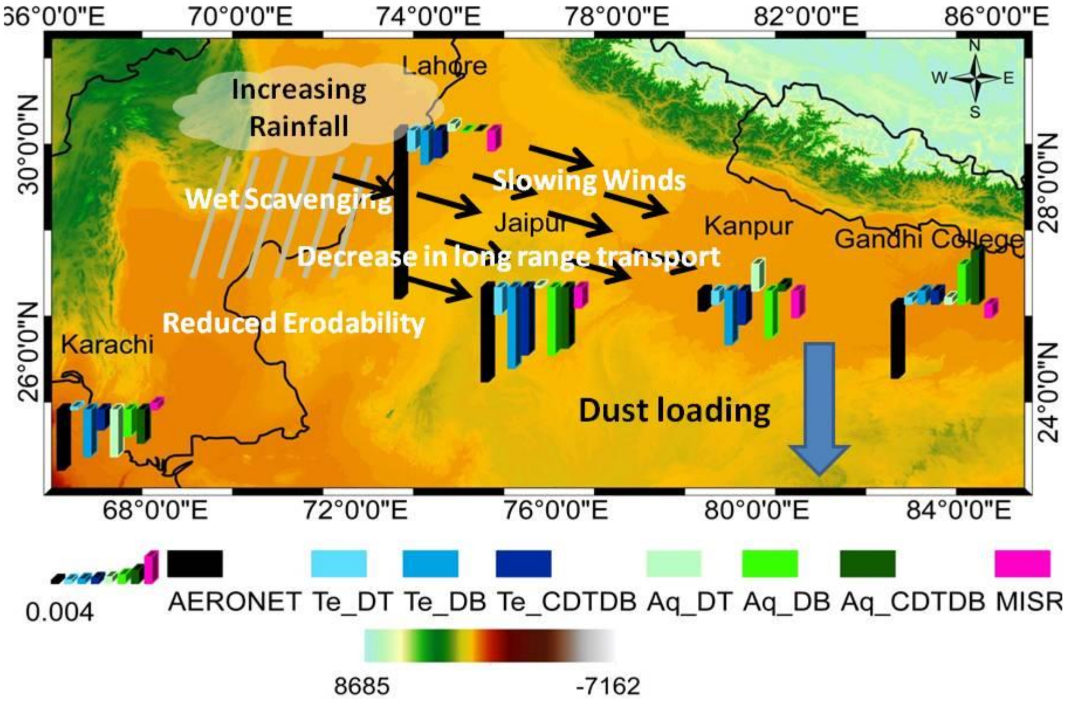


Fig S3: Study region and trend (year$^{-1}$) in AOD at available AERONET sites. Where, Te-Terra and Aq-Aqua, DT-Dark Target, DB-Deep Blue, CDTDB-Combined and DTDB background is depicting elevation (m). The maps are generated with ArcMap(Ver10.2 ) (http://www.esri.com/software/arcgis/arcgis-for-desktop).

## Data details

| Data Source | | Parameter | Data Frequency |
|---|---|---|---|
| AERONET | | Aerosol Optical Depth<br>Fine Mode Fraction<br>Single Scattering Albedo<br>Angstrom Exponent | Daily and All Point Retrievals |
| MODIS | Terra-Dark Target | Aerosol Optical Depth, Angstrom Exponent | Monthly |
| | Terra-Deep Blue | | |

| | | | |
|---|---|---|---|
| | Terra-Combined Dark Target Deep Blue | | |
| | Aqua-Dark Target | | |
| | Aqua-Deep Blue | | |
| | Aqua-Combined Dark Target Deep Blue | | |
| MISR | | Aerosol Optical Depth | Monthly |
| OMI | | UV Aerosol Index | Daily |
| TRMM | | Precipitation | Monthly |
| IMD | | Precipitation | Daily |
| GPCP | | Precipitation | Monthly |
| ECMWF ERA-Interim | | 10m Wind Speed | Daily |
| MERRA2 | | Dust Extinction AOD, Dust Dry Deposition, Dust Wet Deposition | Monthly |
| CALIPSO | | Extinction Coefficient at 532 nm | Monthly |

**References**:


1. Lee, J. *et al.* Characteristics of aerosol types from AERONET sunphotometer measurements. *Atmos. Environ.* **44,** 3110–3117 (2010).
2. Misra, A. *et al.* An overview of the physico-chemical characteristics of dust at Kanpur in the central Indo-Gangetic basin. *Atmos. Environ.* **97,** 386–396 (2014).
3. Dubovik, O. *et al.* Accuracy assessments of aerosol optical properties retrieved from Aerosol Robotic Network (AERONET) Sun and sky radiance measurements. *J. Geophys. Res.* **105,** 9791 (2000).
4. Smirnov, A., Holben, B. N., Eck, T. F., Dubovik, O. & Slutsker, I. Cloud-screening and quality control algorithms for the AERONET database. *Remote Sens. Environ.* **73,** 337–349 (2000).
